# Another Facet to the Anticancer Response to Lamellarin D: Induction of Cellular Senescence through Inhibition of Topoisomerase I and Intracellular Ros Production

**DOI:** 10.3390/md12020779

**Published:** 2014-01-27

**Authors:** Caroline Ballot, Alain Martoriati, Manel Jendoubi, Sébastien Buche, Pierre Formstecher, Laurent Mortier, Jérome Kluza, Philippe Marchetti

**Affiliations:** 1 Inserm U837-JPARC, Université de Lille II, Faculté de Médecine, 1Place Verdun, Lille 59037, France; E-Mails: caro.ballot@gmail.com (C.B.); alain.Martoriati@univ.lille1.fr (A.M.); manel.jendoubi@gmail.com (M.J.); sebastien.buche@chru-lille.fr (S.B.); pierre.formstecher@inserm.fr (P.F.); laurent.mortier@chru-lille.fr (L.M.); jerome.kluza@inserm.fr (J.K.); 2 CBP (Centre de Bio-Pathologie), Banque de Tissus, CHRU (Centre Hospitalier Régional Universitaire), Lille 59037, France

**Keywords:** oxidative stress response, mitochondria, DNA damage, cellular senescence

## Abstract

Lamellarin D (LamD) is a marine alkaloid with broad spectrum antitumor activities. Multiple intracellular targets of LamD, which affect cancer cell growth and induce apoptosis, have been identified. These include nuclear topoisomerase I, relevant kinases (such as cyclin-dependent kinase 2) and the mitochondrial electron transport chain. While we have previously demonstrated that LamD at micromolar range deploys strong cytotoxicity by inducing mitochondrial apoptosis, mechanisms of its cytostatic effect have not yet been characterized. Here, we demonstrated that induction of cellular senescence (depicted by cell cycle arrest in G2 associated with β-galactosidase activity) is a common response to subtoxic concentrations of LamD. Cellular senescence is observed in a large panel of cancer cells following *in vitro* or *in vivo* exposure to LamD. The onset of cellular senescence is dependent on the presence of intact topoisomerase I since topoisomerase I-mutated cells are resistant to senescence induced by LamD. LamD-induced senescence occurs without important loss of telomere integrity. Instead, incubation with LamD results in the production of intracellular reactive oxygen species (ROS), which are critical for senescence as demonstrated by the inhibitory effect of antioxidants. In addition, cancer cells lacking mitochondrial DNA also exhibit cellular senescence upon LamD exposure indicating that LamD can trigger senescence, unlike apoptosis, in the absence of functional mitochondria. Overall, our results identify senescence-associated growth arrest as a powerful effect of LamD and add compelling evidence for the pharmacological interest of lamellarins as potential anticancer agents.

## 1. Introduction

**Figure 1:**
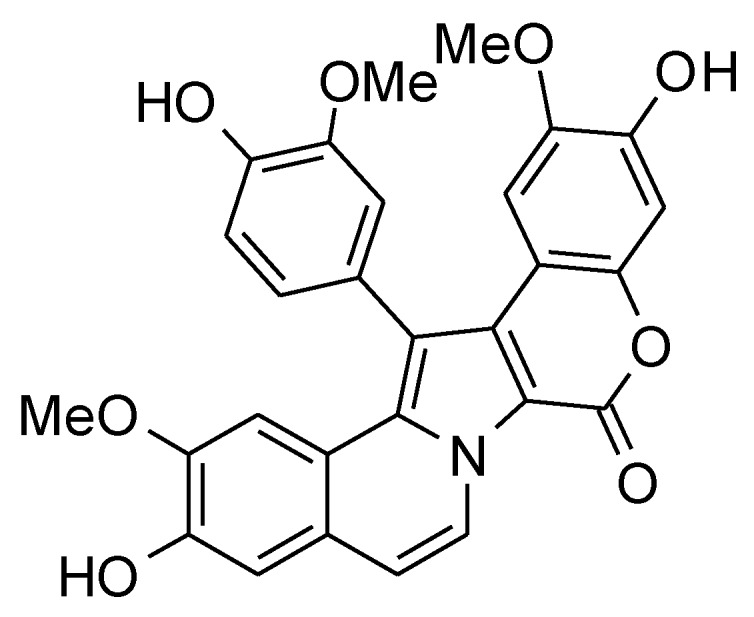
Chemical structure of LamD.

Marine organisms (including invertebrates and microorganisms) constitute a vast reservoir of bioactive molecules exhibiting potential anticancer activities [1,2]. Despite this, several marine-based molecules have reached Phase II and III cancer clinical trials with encouraging results. In addition, Ecteinascidin 743 (*a.k.a* Trabectedin, Yondelis^®^), an antitumor compound extracted from sea quirts and tunicates, has been approved for the treatment of advanced soft tissue sarcomas in European countries [3,4]. Many marine compounds with antitumor effects are natural poisons involved in defense mechanisms and are therefore potentially cytotoxic to cancer cells. For instance, Kahalalide F, a peptide isolated from molluscs, represents a lysosomal poison that promotes non-apoptotic cell death by oncosis in several tumor cells [5]. Despite real advances, in most of cases, mechanisms of anticancer effects of marine-derived drug candidates remain to be determined. Over the past decade, there has been a growing interest in a promising family of natural marine products called lamellarins. Lamellarins were initially isolated from the marine prosobranch mollusc *Lamellaria* sp. and afterward from ascidians (tunicates) that are considered as food supply by *Lamellaria* (for review [6,7]). To date, more than 40 lamellarins, which share a common hexacyclic alkaloid skeleton, have been described [7]. Lamellarins have complex mechanisms of action and exert pleiotropic bioactivities including antiviral properties, antioxidative activities, antiproliferative and cell death-inducing effects (for review [6]). Lamellarin D (LamD) (Figure 1) is undoubtedly one of the most active lamellarins with high potential for cancer therapy [8]. LamD is a multitarget drug endowed with important antitumor activities in a wide variety of cancer cells including multidrug-resistant tumor cell lines [9]. LamD targets several serine/threonine kinases that contribute to the tumorigenesis [10]. Morevoer, LamD preferentially targets cancer cell mitochondria [8], leading to prominent reduction in mitochondrial activity [11]. The mitochondrial targeting depends upon the presence of hydroxyl groups attached to the basic skeleton of LamD [11]. Mitochondrial dysfunctions are observed with micromolar concentrations of LamD and promote rapid mitochondrial permeability culminating in apoptotic cell death [8,11,12]. Thus, there are cumulative evidences that LamD triggers cancer cell apoptosis through direct activation of the canonical intrinsic mitochondrial cell death pathway [8,12]. Interestingly, cytotoxicity of LamD is totally dependent on the presence of functional mitochondria and is dissociated from nuclear signaling pathways such as those dependent on p53 [12]. While the cytotoxic effects of LamD have been largely deciphered [8,11,12], mechanisms and molecular pathways involved in the antiproliferative activities of LamD are still not elucidated. Of note, LamD has also been identified as a potent inhibitor of DNA topoisomerase I [9]. This effect is presumably responsible for the antiproliferative activity observed with sublethal concentrations of LamD [12]. Consequently, in this paper, we investigated the growth inhibition profile of several cancer cell lines exposed to sublethal doses of LamD. Our results add senescence-associated growth arrest to the wide spectrum of anticancer activities of LamD and indicate that LamD-induced senescence is largely dependent on the effect of LamD on topoisomerase I and ROS generation.

## 2. Results and Discussion

### 2.1. Lamellarin D Induces Senescence-Like Growth Arrest in Cancer Cells

**Figure 2:**
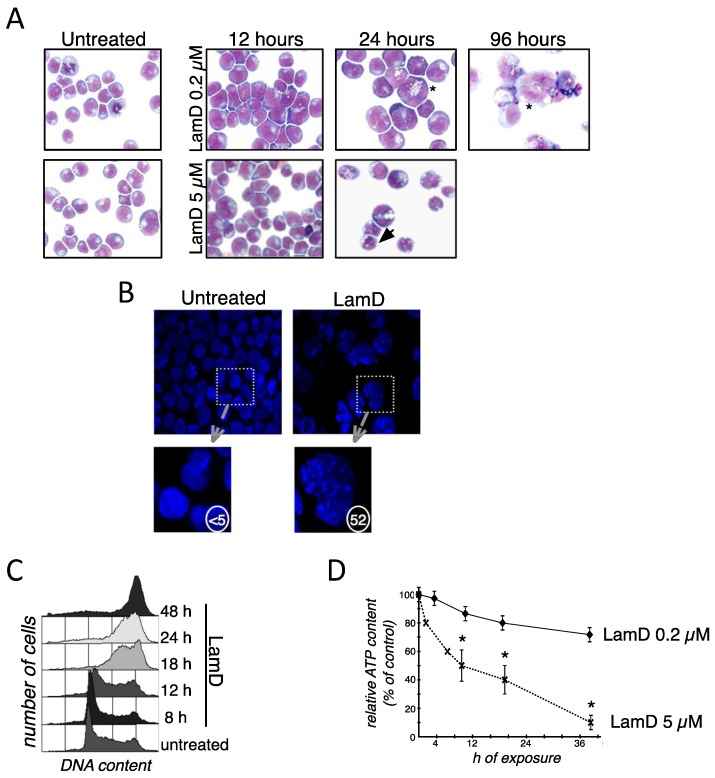
Senescence-like growth arrest in cancer cells exposed to LamD. (**A**) May-Grunwald Giemsa staining of P388 cells exposed to 5 µM or 0.2 µM LamD for the indicated times. Arrows show apoptotic phenotype and * show senescence-like phenotype; (**B**) examination of DAPI-stained nuclei in P388 cells exposed to 0.2 µM LamD for 24 h. Original magnification ×400. Numbers indicate the percentage of cells displaying DNA damage foci; (**C**) cell cycle distribution in P388 cells exposed to 0.2 µM LamD for the indicated times; (**D**) kinetics of the effects of LamD (0.2 µM or 5 µM) on ATP levels in P388 cells; * *p* < 0.05 between two groups.

**Figure 3:**
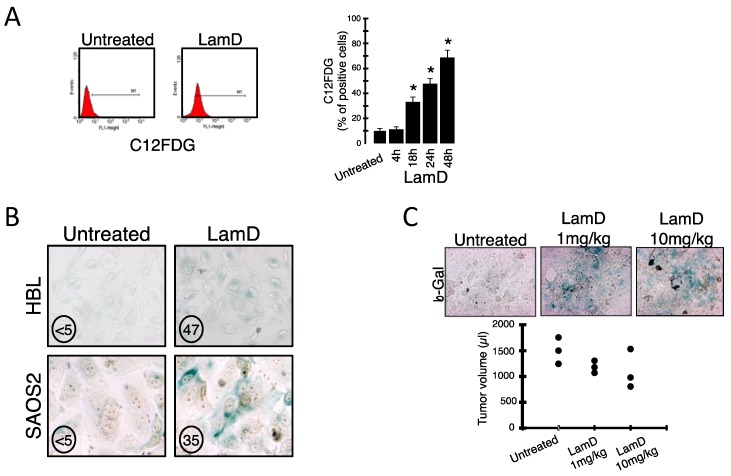
(**A**) (*left*) Representative flow cytometric profiles of senescent P388 cells treated with 0.2 µM LamD for 24 h (or kept untreated) then stained with C12FDG, a fluorogenic substrate for SA-β-galactosidase before analysis; (*right*) Kinetics of the effects of 0.2 µM LamD on senescence of P388 cells assessed by flow cytometry after C12FDG staining, * *p* < 0.05 between two groups; (**B**) senescence was assessed in human HBL melanoma cells and SAOS2 osteosarcoma cells upon LamD exposure (20 nM for 72 h) detecting SA-β-galactosidase by cytochemistry; Numbers indicate the percentage of positive cells; (**C**) representative histological sections from tumors of HBL-injected SCID mice treated or not with the indicated doses of LamD for 2 weeks then stained for detection of SA-β-gal as in F and tumor volumes were measured (3 mice per group).

We aimed to determine the phenotype of several cancer cells exposed to sublethal concentrations of LamD [8,12]. While 5 µM LamD mainly reduced cell viability by induction of an apoptotic phenotype, morphological examination of P388 leukemia cells exposed to 0.2 µM LamD revealed growth arrest with the appearance of “giant” cells over time (Figure 2A). LamD-induced cell enlargement was associated with vacuolated cytoplasm, the presence of enlarged nuclei and sometimes multiple nuclei (Figure 2A). In addition, DAPI-stained nuclei appeared enlarged with the presence of multiple foci (Figure 2B), a feature consistent with the presence of DNA damage [13]. Confirming previous observations [9,12], cell cycle analysis indicated that 0.2 µM LamD induced a transient S-phase blockage (>12–18 h of exposure) followed by an accumulation of cells in G2/M phase (Figure 2C). The quasi-totality of cells was arrested in G2/M phase upon 48 h of treatment. Since microscopic examination revealed mitoses only infrequently (Figure 2A), the presence of enlarged cells observed after LamD exposure probably corresponded to cells arrested at the G2 checkpoint and not during mitosis. In an attempt to determine if growth-arrested cells remained metabolically active, determination of intracellular ATP level was performed in P388 cells exposed to LamD. Contrasting with the effect observed at higher concentrations, 0.2 µM LamD, a dose that induced leukemia growth arrest (Figure 2A,C), did not profoundly curtail intracellular ATP production (Figure 2D). Since the above morphological changes were reminiscent of cellular senescence [14,15], this prompted us to analyze the presence of senescence-associated β-galactosidase (SA-β-gal), the typical marker of senescent cells [16], in LamD treated cells. As shown in Figure 3A, LamD exposure caused the time-dependent induction of SA-β-gal activity as determined by flow cytometry in P388 cells stained with the fluorescent β-galactosidase substrate, C12FDG (Figure 3A). To confirm this result, we also subjected other cancer cell lines (such as the human melanoma cell line HBL and the human osteosarcoma SAOS2) to sublethal concentrations of LamD. Strikingly, the cytochemical assessment of SA-β-gal also revealed significant increase in the senescent marker after treatment with LamD (Figure 3B). Like LamD-treated P388 cells, HBL and SAOS2 cells positive for SA-β-gal were enlarged (Figure 3B). As shown in Figure 3C, LamD also induced the apparition of SA-β-gal activity *in vivo* in HBL melanoma tumor model in SCID mice [17]. In this preclinical tumor model, treatment with LamD reduced tumor growth (Figure 3C). Altogether, these results indicate that LamD is an inducer of senescence-associated growth arrest in several cancer cells.

### 2.2. Senescence-Associated Growth Arrest Induced by Lamellarin D Is Dependent on Its Effect on Topoisomerase I

**Figure 4:**
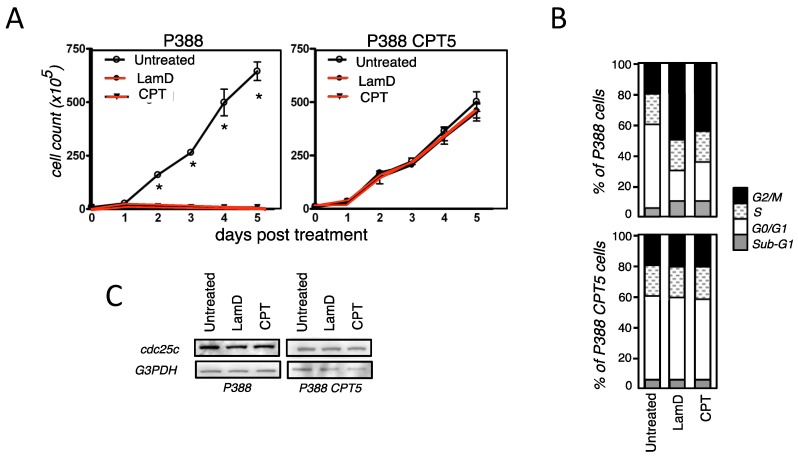
Senescence-like growth arrest depends on the effects of LamD on topoisomerase I (**A**) P388 cells and P388CPT5 cells were incubated in the presence of 0.2 µM LamD, 0.2 µM camptothecin (CPT) or kept untreated, then cells were counted every day. Data are means ± SD of three independent experiments. * *p* < 0.05 between two groups; (**B**) cell cycle analysis of P388 and P388CPT5 cells exposed to 0.2 µM LamD or CPT for 24 h. Data are representative of at least four independent experiments; (**C**) immunoblot analysis of cdc25c protein expression in P388 and P388CPT5 cells exposed to 0.2 µM LamD or CPT for 18 h. G3PDH was used as loading control. Data are from one representative of two independent experiments.

**Figure 5:**
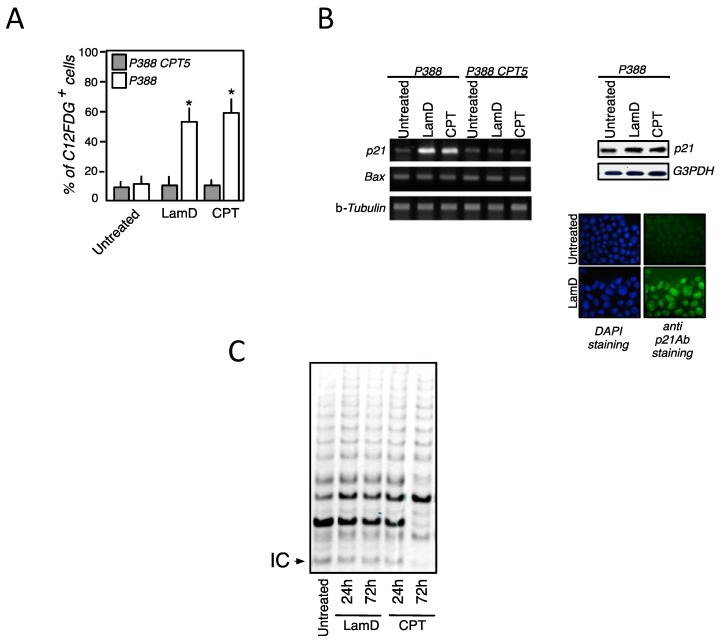
(**A**) Analysis of senescence by flow cytometry after C12FDG staining of P388 and P388CPT5 cells treated with 0.2 µM LamD or CPT. Data are means ± SD of four independent experiments. * *p* < 0.05 compared to untreated group; (**B**) LamD, like CPT, induced the expression of P21 in P388 cells. (*left*) RT-PCR analysis of P21 and Bax mRNA expression in P388 and P388CPT5 cells treated with 0.2 µM LamD or CPT for 18 h. β-tubulin was used as control. Data are representative of two independent experiments; (*upper right*) immunoblot analysis of P21 protein expression in P388 cells treated as above. G3PDH served as control; (*lower right*) immunofluorescence analysis of P21 protein expression in P388 cells treated as above then counterstained with DAPI before analysis under the microscope. Data are representative of two independent experiments; (**C**) P388 cells were exposed to 0.2 µM LamD or CPT then at indicated times assayed for telomerase activity using the TRAP assay. IC refers to the 36-bp internal control band.

DNA topoisomerase I has been the first identified target of LamD [9]. LamD has been unveiled as a potent inhibitor of topoisomerase I responsible for DNA damage, p53 activation and growth inhibition [9,12]. In contrast, we have previously reported that the cytotoxic activity of LamD is independent on topoisomerase I [12]. Indeed, high concentrations of LamD maintained a sustained level of apoptosis in the topoisomerase I-mutated cells (P388CPT5) that are resistant to cell death induced by the prototype inhibitor of topoisomerase I, camptothecin (CPT) [8]. Here, we addressed the question whether the inhibition of topoisomerase I could be involved in the senescence-associated growth arrest induced by sublethal concentration of LamD. First, we studied the apparition of growth arrest in P388CPT5 upon LamD exposure and used camptothecin as control (Figure 4A). Contrasting what was observed in P388 cells, kinetic studies revealed that LamD, as well as CPT, was devoid of any antiproliferative effect in P388CPT5 (Figure 4A). Confirming these results, LamD and the prototype topoisomerase I inhibitor CPT arrested P388 cells in G2 phase, a situation not observed in P388CPT5 (Figure 4B). G2 arrest was associated with a decline in the protein level of the phospatase Cdc25c in P388 cells exposed to drugs. Once again, this effect was absent in the topoisomerase I-mutated cells exposed to LamD or CPT (Figure 4C). Besides, growth arrest was associated with the induction of cellular senescence in P388 cells treated with LamD as deemed by the percentage of C12FDG+ cells (Figure 5A). A nearly identical percentage was observed for P388 cells after treatment with CPT. Conversely, LamD, as well as CPT, did not elicit any SA-gal activity in the topoisomerase I-mutated cells (Figure 5A). Of note, the rate of senescence induced by LamD was comparable to that observed after CPT exposure (Figure 5A). The CDK inhibitor, p21, can be considered as a senescence marker that links cell cycle arrest and cellular senescence. Additionally, evidence shows that CPT induced the expression of p21 in colon cancer cells, and this expression is required for senescence [18]. Confirming these data, LamD, as well as CPT, induced p21 mRNA expression exclusively in wild-type P388 cells. Western blot analysis confirmed the p21 increase at the protein level, and immunofluorescence data revealed its nuclear localization in P388 treated with LamD (Figure 5B). Taken together, these results suggest that senescence-like growth arrest induced by LamD is strictly dependent on its inhibition of DNA topoisomerase I.

Genotoxic agents such as conventional chemotherapeutic agents induce senescence by different mechanisms and some of them have been associated with suppression of telomerase activity [19]. We evaluated telomerase activity in cells exposed to LamD using the PCR-based telomeric repeat amplification protocol. LamD treatment of P388 cells did not result in an important reduction in telomerase activity within the first 24–72 h of drug exposure. No effect was observed with CPT for 24 h. Thus, telomere shortening probably does not constitute the principal mechanism resulting in senescence induced by LamD.

### 2.3. Lamellarin D Induced Extra-Mitochondrial ROS Accumulation in Senescent Cells

**Figure 6:**
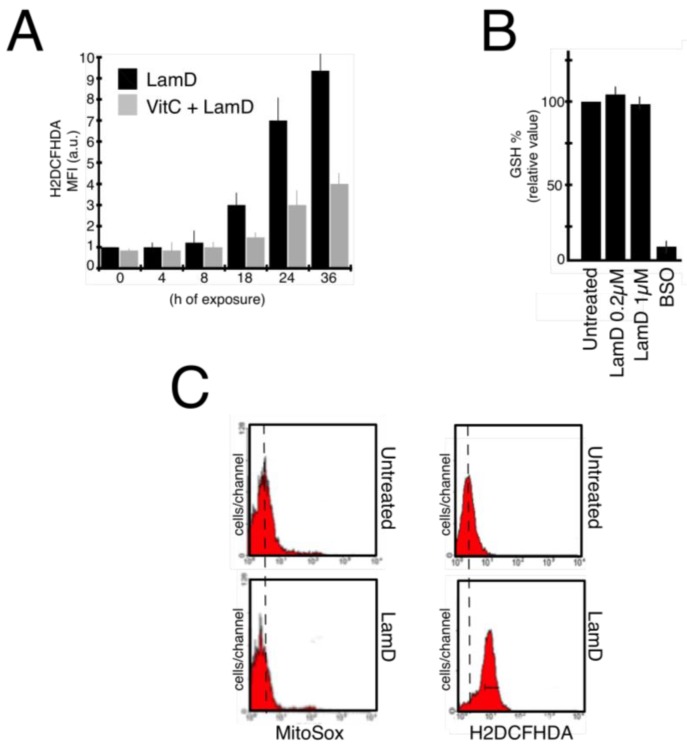
Intracellular generation of ROS in cancer cells treated with LamD (**A**) P388 cells were incubated with 0.2 µM LamD alone or in association with 100 µM Vitamin C (VitC) and at indicated times, cells were stained with H2DCFHDA before flow cytometric analysis; (**B**) P388 cells were either kept untreated or exposed to LamD at indicated doses for 24 h then GSH content was determined by flow cytometry. As control, cells were treated with 10 mM BSO for 12 h to deplete intracellular GSH. Results are expressed as fluorescence as a percentage of untreated cells (mean ± SD of three independent experiments in triplicates); (**C**) representative flow cytometric profiles of P388 cells treated with 0.2 µM LamD for 36 h (*lower panels*) or kept untreated (*upper panels*) then stained with either H2DCFHDA (*right panels*) or MitoSox (*left panels*) before analysis.

**Figure 7:**
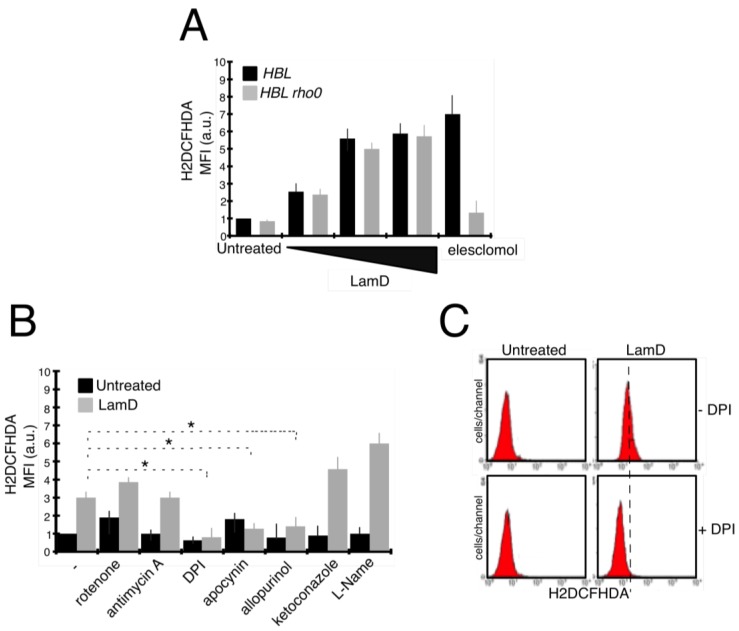
(**A**) HBL and HBL ρ0 cells were incubated with increasing doses of LamD (0.05 µM, 0.2 µM, 0.5 µM) for 24 h. As control of the absence of functional mitochondria in HBL ρ0, cells were incubated with the mitochondrial oxidative agent, eleclomol (300 nM for 18 h). ROS generation was detected by flow cytometrica analyses of H2DCFHDA fuorescence. Data are means ± SD of two experiments in triplicates; (**B**) P388 cells were kept untreated or treated with 0.2 µM LamD for 24 h in the presence or absence of one of the following ROS inhibitors; 1 µM Rotenone, 1 µM Antimycin A, 10 µM DPI, 250 µM Apocynin, 1mM Allopurinol, 10 µM Ketoconazol, 10mM L-NAME. Then, cells were stained with H2DCFHDA before analysis. Data are means ± SD of three independent experiments, * *p* < 0.05; (**C**) flow cytometric profiles of H2DCFHDA fluorescence in HBL cells exposed to 0.2 µM LamD for 18 h in the presence or absence of 100 µM DPI. Profiles are representative of two independent experiments in duplicates.

Recent studies have suggested that reactive oxygen species (ROS) can mediate senescence induced by anticancer drugs [19]. We first studied the pro-oxidant cell response to LamD. The generation of ROS in P388 cells was determined by flow cytometry with the general redox-sensitive fluorescent dye, H2DCFHDA. As shown in Figure 6A, LamD stimulated the intracellular generation of ROS over time, beginning after 18 h of exposure concomitantly withthe apparition of senescence-like growth arrest (Figure 2 and Figure 3). LamD-induced ROS was limited by the pre-incubation with the antioxidant vitamin C (Figure 6A). A similar increase in ROS was observed in HBL cells upon LamD exposure (Figure 6D). ROS increase was not accompanied by changes in GSH content in LamD-treated P388 cells (Figure 6B). Since mitochondrial electron transport chain is considered the principal source of intracellular ROS [20], we examined whether LamD could promote mitochondrial ROS production detected with the mitochondrial anion superoxide detector, MitoSox. Under conditions in which LamD significantly increased H2DCFHDA fluorescence, MitoSox fluorescence was not affected by exposition to LamD (Figure 6C). To confirm the existence of extra-mitochondrial sources of LamD-induced ROS, we used the partially respiration-deficient cell line HBL ρ0 that produces less mitochondrial ROS than its parental cell line, HBL [21]. Unlike the mitochondrial pro-oxidant, elesclomol, which promoted ROS quasi-exclusively by active mitochondria [21], LamD induced about the same extent of ROS production in HBL and HBL ρ0 (Figure 7A), indicating that LamD can induce ROS in absence of intact electron transport chain and confirming that mitochondria are not the major source of ROS upon LamD exposure. To get insight into the potential sources of ROS induced by LamD, we incubated P388 cells with various inhibitors of ROS-producing enzymes before treatment with LamD then ROS generation was evaluated after H2DCFHDA staining (Figure 7B). LamD-induced ROS was significantly inhibited by diphenylene iodonium (DPI), a classical inhibitor of flavoenzymes, by apocynin, a more specific inhibitor of NADPH oxidase and by allopurinol an inhibitor of xantine oxidase activity. In contrast, the mitochondrial ROS inhibitors, rotenone and antimycin A did not prevent ROS generation by LamD. No effect was observed with the inhibitor of NOS, L-NAME or with the inhibitor of cytochrome P450, ketoconazole. Interestingly, a strong inhibitory effect of DPI on LamD-induced ROS generation was also observed in HBL cells (Figure 7C). Altogether, the aforementioned data indicate that LamD generates extra-mitochondrial ROS in cancer cells. 

### 2.4. Role of ROS Production in Senescence and DNA Damage Induced by LamD

**Figure 8:**
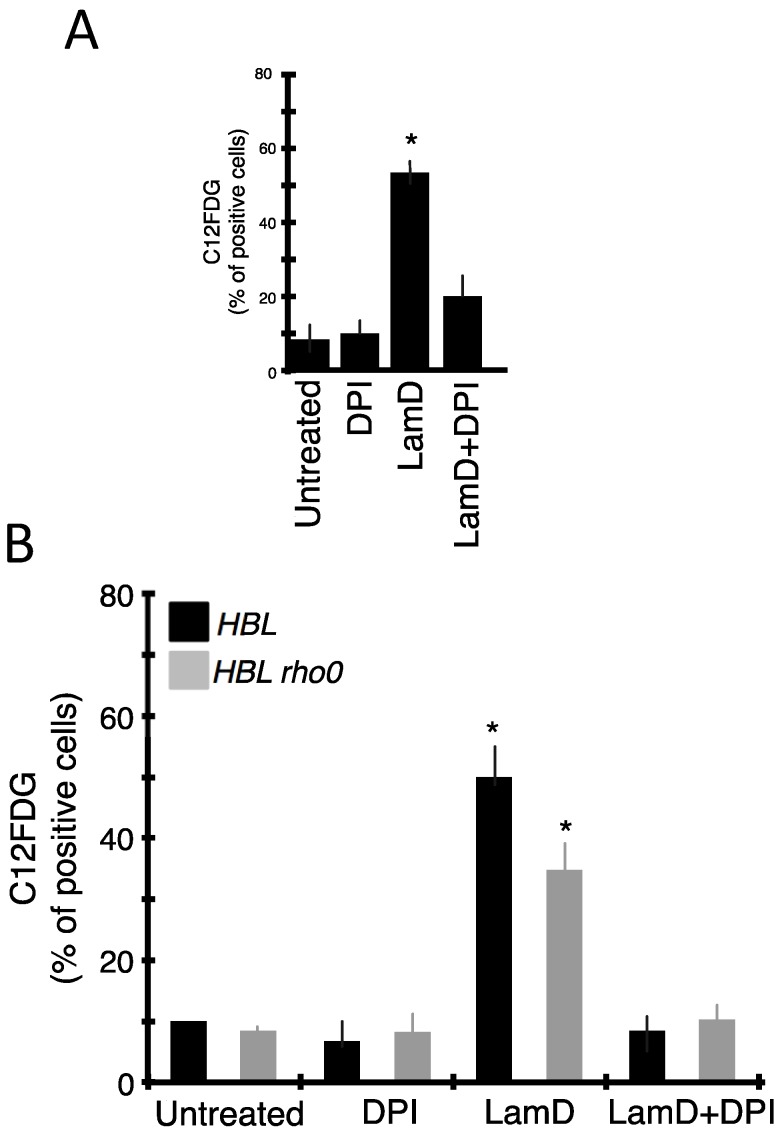
LamD-induced ROS generation participates in the occurrence of the senescent phenotype. P388 cells (**A**) or HBL and HBL ρ0 cells; (**B**) were incubated with 0.2 µM LamD alone or in association with 10 µM DPI for 24 h then stained with C12FDG before flow cytometric analysis. Data are means ± SD of three independent experiments. * *p* < 0.05 compared to control.

**Figure 9:**
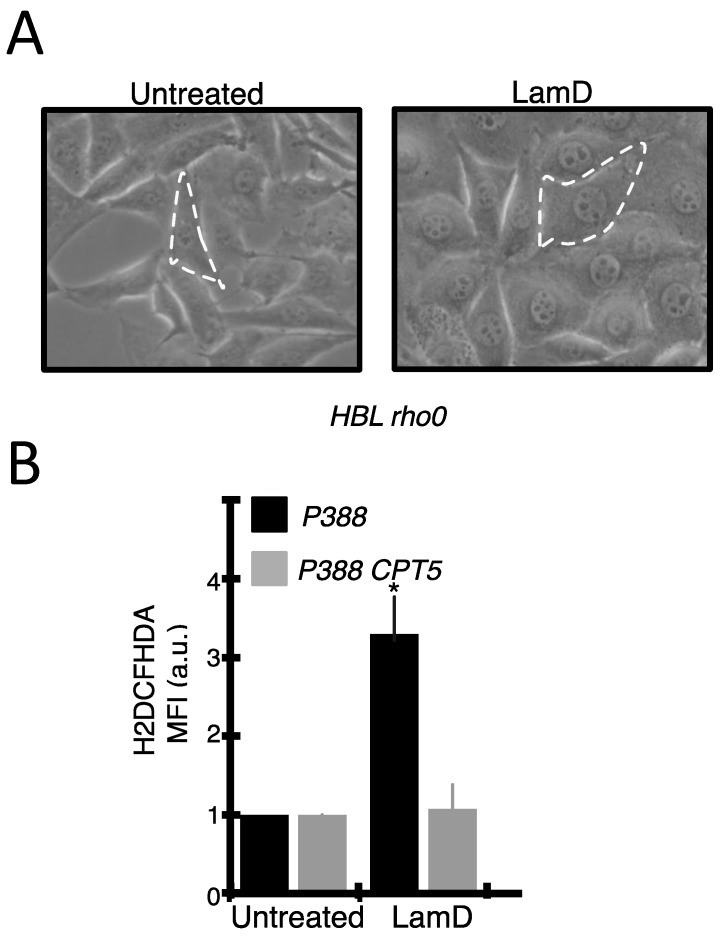
(**A**) Phase contrast microscopy analysis of HBL ρ0 cells exposed to 0.2 µM LamD for 36 h. (original magnification ×400). Dashed lines delineate representative cells. Note the presence of enlarged cells after LamD exposure; (**B**) P388 and P388 CPT5 cells were incubated with 0.2 µM LamD for 24 h then stained with H2DCFHDA before flow cytometric analysis. Data are means ± SD of two independent experiments in duplicates; * *p* < 0.05 compared to control.

We next studied whether the generation of ROS can contribute to the senescent phenotype induced by LamD (Figure 8). Pre-treatment of P388 or HBL cells with DPI remarkably minimized the LamD-induced apparition of the senescent marker, SA-β-gal (Figure 8A,B), also as it prevented the generation of ROS (Figure 6 and Figure 7). Moreover, HBL ρ0 cells, which responded to LamD exposure by increasing intracellular ROS (Figure 6 and Figure 7), displayed a classical senescent phenotype (demonstrated by significant increase in C12FDG positive cells (Figure 8B) and the presence of typical morphological changes (Figure 9A)). Accordingly, upon LamD exposure, the topoisomerase I-mutated cell line, P388 CPT5, which was resistant to the apparition of senescence (Figure 2 and Figure 3), did not produce intracellular ROS (Figure 9B). All these data underscore the strict correlation between ROS production and senescence in LamD-treated cells indicating that LamD can promote the senescent phenotype through ROS generation.

**Figure 10:**
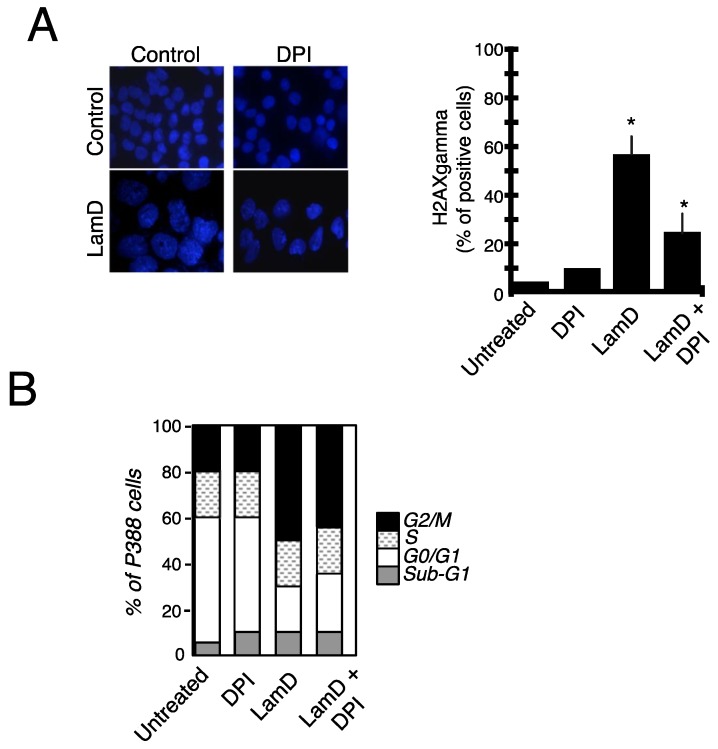
Links between ROS generation and DNA damage induced by LamD (**A**) *right* Morphological examination of DAPI-stained nuclei in P388 cells exposed to 0.2 µM LamD alone or in the presence of 10 µM DPI for 24 h (original magnification ×400); *left* P388 cells were treated as above then H2AXgamma positive cells were detected by flow cytometry as described in materials and methods * *p* < 0.05 compared to control; (**B**) cell cycle distribution in P388 cells exposed to LamD and/or DPI as above.

Finally, we investigated the role of ROS in DNA damage induced by LamD (Figure 10). Exposure of P388 cells to DPI at concentrations that inhibited ROS production (Figure 7A) partially reduced the onset of DNA damage induced by LamD as estimated by nuclear foci and the detection of the phosphorylated form of histone H2AX (H2AXγ), a good indicator of double-strand DNA breaks (Figure 10A). Moreover, DPI had only minimal protective effects on LamD-induced G2 arrest (Figure 10B). 

### 2.5. Proposed Mechanisms of LamD-Induced Senescence

LamD is an uncommonly potent anticancer agent that displays cytotoxic effects against a large panel of cancer cell lines and tumor xenografts [8,9]. The anticancer effect of LamD results from its unique mitochondrial effect leading to cancer cell death [11].

Here, we demonstrated that LamD induced cellular senescence at sublethal doses correlating with IC50 value for antiproliferative activity [9,12]. Cellular senescence is a state of cell cycle arrest +ly triggered by telomere shortening (*a.k.a.* replicative or mitotic senescence) or by intracellular stresses (*a.k.a.* accelerated or premature senescence). Thus, senescence of cancer cells can occur in response to DNA damaging agents and results in proliferation arrest associated with a specific morphological and biochemical phenotype (for review [19]). Herein, we provided strong evidence for adding LamD to the list of senescence-inducing agents including many chemotherapeutic agents such as topoisomerase I and II inhibitors [19]. Thus, it has been observed that the major cellular response to nonlethal concentrations of the topoisomerase II inhibitor, doxorubicin, was senescence observed in almost all tumor cell lines *in vitro* [22] as well as *in vivo* in human tumors xenografted in nude mice and even in biopsies from patients [23]. We observed that LamD provokes growth arrest in the G2 stage of the cell cycle sustained by the increased expression of the cyclin-dependent kinase inhibitor, p21 [24]. This is accompanied by a distinguishable morphological phenotype consisting of flattened and enlarged cells with cytoplasmic granules and a large nucleus associated with increased SA-β-gal staining. However, the absence of major alteration in telomerase activity suggests that LamD caused DNA damage- and stress-induced senescence rather than the classical replicative senescence [25]. Interestingly, senescence is considered as a major antiproliferative response that determines treatment outcome in apoptosis-resistant cancer [23]. Senescence can be viewed as a tumor-suppressive mechanism and therefore efficient senescence-inducing agents, like LamD, could have a significant advantage in the clinical treatment of cancer. Cellular senescence may represent an alternative outcome to anticancer therapies, and senescence, which requires much less concentrations of drugs, may be associated with fewer or less severe side effects than cytotoxicity [19].

Our results also shed light on the potential mechanisms associated with the senescence-associated growth arrest induced by LamD. Firstly, we demonstrated a requirement for topoisomerase I in the senescence response to LamD. Moreover, LamD can produce significant levels of single [9] and double strand breaks (DSB) as shown by the presence of H2AX phosphorylation. This is in agreement with the senescence observed with campthotecin (another topoisomerase I inhibitor) in colon cancer [18]. Treatment of cells with nonlethal concentrations of camptothecin induced limited DNA damage, then p53 activation, which in turn up-regulated the expression of p21 [18]. This is in line with the effects of LamD on p21 expression (Figure 6 and Figure 7), suggesting that the cellular senescence response to topoisomerase I inhibitors depends on p21. Secondly, we also demonstrated that ROS are required for senescence induced by LamD. Many anticancer drugs generate excessive intracellular ROS that participate in their cytotoxic effects [26]. Furthermore, senescent cells caused by chemotherapeutic agents also exhibited a significantly higher level of ROS [27]. The significant contribution of oxidative stress to premature senescence was confirmed by the observations that inhibition of ROS largely attenuated senescence induced by anticancer agents [28]. Mitochondria are considered to be the major source of intracellular ROS. Surprisingly, pharmacological evidences presented herein suggest an extra-mitochondrial origin of ROS in LamD-induced senescence. Since the onset of senescence was sensitive to the NADPH oxidase (NOX) inhibitor, DPI, one can envisage that NOX-derived ROS may be involved in LamD-induced senescence. There are growing evidences that enzymes of the NOX family play an important role in the redox signaling in healthy and tumor cells [29]. Consistent with our data, the NADPH oxidase, NOX4, has been identified as a good candidate for ROS production in senescent cells [30]. Interestingly, NOX4-dependent ROS also regulated P21 expression [30].

**Figure 11:**
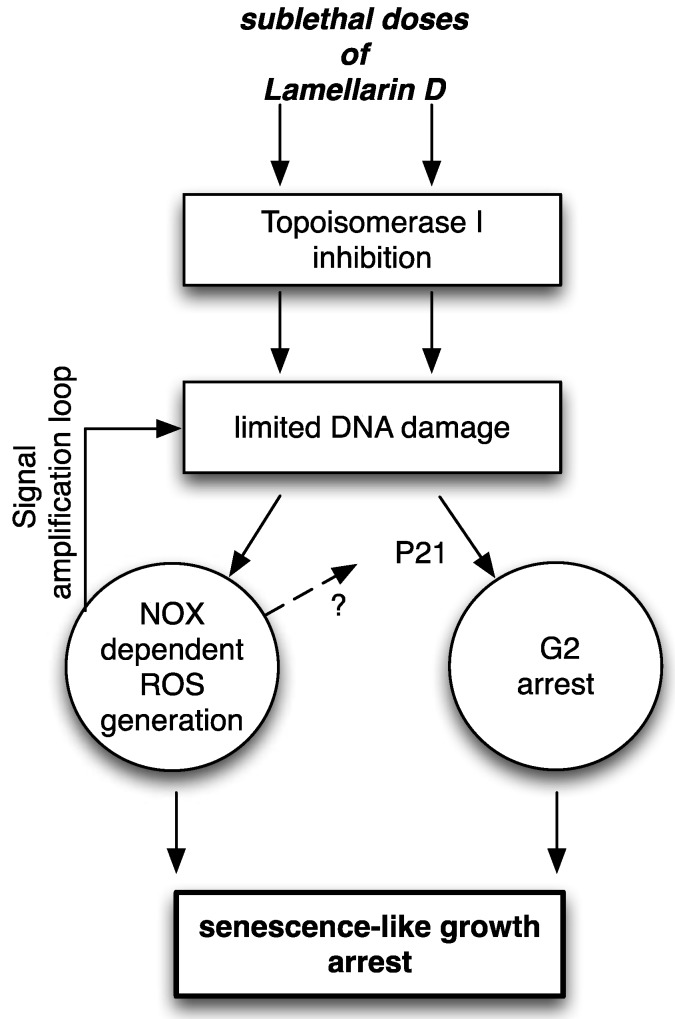
Proposed mechanisms of senescence-like growth arrest induced by LamD. (See text for details).

How are ROS and DNA damage interconnected in LamD-induced senescence? Typically, increased ROS generation can trigger oxidative DNA damage then creates DNA DSB leading to premature senescence. Accordingly, radiotherapy can induce senescence in cancer cells through ROS-mediated DNA damage [31]. However, it is likely that induction of senescence by LamD does not follow this typical cascade. Indeed, we observed that the protection of DPI on LamD-induced DNA damage was only partial. Moreover, kinetic studies indicate that DNA damage induced by LamD occurred before the onset of ROS ([12] and data not shown). Moreover, in topoisomerase I-mutated cells, no increase of the level of ROS was observed upon LamD exposure suggesting that DNA damage caused by LamD resulted in the induction of ROS. Similarly, it has been demonstrated that DNA damage can induce ROS generation, which is fully prevented by pre-incubation of cells with DPI, through activation of the H2AX NOX1/Rac1 pathway, [32]. However, we cannot exclude other mechanisms of regulation and, of note, our results suggest the existence of a signal amplification loop involving ROS and DNA damage that may favor the persistence of LamD-induced DNA damage (Figure 11). Finally, based on above evidences and previous data [8,9,12], one can envision the speculative multistep scenario forcing LamD-treated cells toward senescence-associated growth arrest (Figure 11): (i) sublethal doses of LamD inhibit topoisomerase I and induce limited DNA damage; (ii) DNA damage activates NOX leading to ROS generation; (iii) DNA damage activates p21 and provokes G2 growth arrest. All these events contribute to the onset of morphological and biochemical signs of senescence.

## 3. Experimental Section

### 3.1. Chemicals

Lamellarin D was synthesized at Pharmamar (Madrid, Spain). LamD was initially dissolved in DMSO at 10 mM. Drug stock solution was aliquoted, kept at −20 °C and freshly diluted with PBS to the desired concentration immediately before use. All fluorochromes including 4′,6-diamidino-2-phenylindole (DAPI), 5-dodecanoylaminofluorescein di-β-d-galactopyrano-side (C12FDG), 6-carboxy-2′,7′-dichlorodihydrofluorescein diacetate (H2DCFHDA) and MitoSoxRed reagent were purchased from Life Technologies, Grand island, NY, USA. Other reagents were purchased from Sigma-Aldrich (St. Louis, MO, USA) unless otherwise stated.

### 3.2. Cell Culture

The mouse leukemia cell line P388, its topoisomerase I-mutated subclone (P388CPT5) resistant to camptothecin ([33], a gift from JF Riou, Rhône-Poulenc Rorer, France) and the HBL cutaneous melanoma cell [8,9,12] line (kindly provided by Pr. G. Ghanem, Universite Libre de Bruxelles, Institut Jules Bordet, Bruxelles, Belgium) were routinely cultured in RPMI 1640 medium (Invitrogen, Carlsbad, CA, USA) supplemented with l-glutamine, antibiotics and 10% heat-inactivated FCS. We have also generated human melanoma cells lacking mitochondrial DNA (HBLρ0) from the HBL cell line based on standard protocols [11].

### 3.3. Microscopic Analysis of Cells

Morphological examination was performed after May Gruenwald Giemsa staining and visualization of SA-β-gal activity was detected following the classical cytochemical protocol [34]. For fluorescence microscopy, P388 cells were fixed in 4% paraformaldehyde for 10 min then permeabilized with 0.1% Triton X-100 for other 10 min. After washing twice with PBS + 2% FCS, cells were incubated with the monoclonal anti-p21 antibody (1:250; F-5, SantaCruz Biotechnology Inc., Santa Cruz, CA, USA) on ice overnight. After three washes in PBS, cells were incubated with AlexaFluor488-conjugated antibody (1:800; Life Technologies) for 1 h at room temperature. Cells were counterstained with 4′,6-diamidino-2-phenylindole (DAPI, 5 mg/mL) to label nuclear DNA. All samples were viewed using a Leica DMR microscope.

### 3.4. Flow Cytometric Analysis

For cell cycle determination, cells were fixed overnight at 4 °C using 70% ice-cold ethanol-PBS followed by propidium iodide (50 µg/mL) staining and cell cycle data were analyzed using the relevant Cell Cycle Analysis software (FlowJo, Tree Star, Ashland, OR, USA). For measurement of SA-β-gal activity, cells were incubated with a non-fluorescent substrate of SA-β-gal, C12FDG at 30 µM, washed, and the level of green fluorescent product was detected by flow cytometry as described [13,34]. Detection of ROS was assessed with several oxidation-sensitive fluorescent probes such as mitochondria-targeted hydroethydine (Mito-SOX, 2.5 µM for 20 min at 37 °C) and H2CFHDA (50 nM for 20 min at 37 °C) following classical protocols [17]. For evaluation of double strand breaks, the H2AX Phosphorylation Assay kit (Upstate, Lake Placid, NY, USA) was used according to previous reports [35]. Cells were stained with the anti-phospho-histone H2AX-FITC conjugate, which recognizes H2AX phosphorylated at serine 139, or with normal mouse IgG conjugate used as control. All samples were analyzed on a FACS Canto II cytofluorometer (Beckton Dickinson, Franklin Lakes, NJ, USA).

### 3.5. Determination of Intracellular Glutathione

The total glutathione content was determined with the glutathione sensitive probe, monochlorobimane (40 µM for 30 min) as described [12]. Fluorescence was measured at 460 nM using a microplate-reading fluorometer (Fluorocount, Packard Instrument Company, Meriden, CT, USA) with excitation at 360 nM.

### 3.6. RT-PCR Analysis

Total RNA was isolated with the TRIzol reagent (Invitrogen, Carlsbad, CA, USA) then cDNA was synthesized as previously described [36]. RT-PCR experiments were performed using P21 forward primers (5′-GGAGCAAAGTGTGCCGTTGTC-3′) and P21 reverse primers (5′-GAGGAAGTACTGGGCCTCTTG-3′); Bax forward primers (5′-GGCGAATTGGAGATGAACTGG-3′) and Bax reverse primers (5′-GCTAGCAAAGTAGAAGAGGGC-3′). The primer set used to amplify β-tubulin was: Forward 5′-CAACGTCAAGACGGCCGTGTG-3′ and reverse: 5′ GACAGAGGCAAACTGAGCACC-3′. The cycling conditions were as follows: 94 °C for 4 min, then 30 cycles consisting of 30 s at 94 °C, 30 s at 58 °C, and 1 min at 72 °C. RT-PCR reaction mixtures were electrophoresed on 2% agarose gel stained with ethidium bromide.

### 3.7. Immunoblot Analysis

Twenty micrograms of proteins of cell extracts were subjected to 12% SDS-PAGE and transferred onto nitrocellulose membranes (Amersham Life Science, Buckinghamshire, UK) which were probed with antibodies against cdc25c (1:1000, C-20 sc-327; SantaCruz Biotechnology Inc., Santa Cruz, CA, USA), or against p21 (1:1000; F-5, SantaCruz Biotechnology Inc.). Protein loading was checked using a rabbit anti-G3PDH (1:1000; Trevigen, Gaithersburg, MD, USA) antibody. Primary antibodies binding was then detected with secondary horseradish peroxidase-conjugated-specific antibodies (1:1000; Biorad, Hercules, CA, USA) and visualized by enhanced chemiluminescence following the manufacturer’s protocol (Amersham Pharmacia Biotech, Piscataway, NJ, USA).

### 3.8. Measurement of Intracellular ATP

One hundred and five cells/mL were incubated with LamD for the indicated times then cells were washed with PBS for bioluminescent ATP content evaluation with the cell-titer Glow assay kit (Promega, Madison, WI, USA) following manufacturer’s instructions.

### 3.9. Telomerase Repeat Amplification Protocol (TRAP) Assay

To determine telomerase activity, the TRAPeze kit (Intergen, Purchase, NY, USA) was used in agreement with manufacturer’s recommendations as previously depicted [37].

### 3.10. *In Vivo* Experiments

All procedures with animals were conducted in accordance with the Institutional guidelines following the procedure detailed elsewhere [17]. Briefly, nine female severe combined immunodeficient (SCID) mice were injected with 1 × 10^6^ HBL cells, mixed (1:1 volume) with BD Matrigel Basement Membrane Matrix. When tumors reached a palpable volume, mice were ramdomly divided into three groups: one control group mice were treated with PBS with the same schedule as the treated animals; two LamD groups with mice treated with LamD at 10 mg/kg or 1 mg/kg (i.v. injection for 5 day/week). After two weeks of treatment, mice were sacrificed, and tumors were measured and removed for histological analysis. 

### 3.11. Statistics

Data were evaluated using GraphPad program (GraphPad Software, San Diego, CA, USA). Data are presented as the mean ± SD. The student’s *t*-test was used to compare data sets. Statistical significant differences were determined for *p* values of less than 0.05.

## 4. Conclusions

In essence, our findings establish the marine drug, LamD, as a potential pro-senescent therapy against cancer and define the senescence program as a cellular stress response elicited by LamD. These results should reinforce our therapeutic interest for the lamellarin family of marine natural products.
